# Personal and Surgical Reminiscences of the War*Abstract of the Orator’s Address before the Survivors’ Association of Ex-Confederate Surgeons, at Spartanburg, S. C., April 22, 1896.

**Published:** 1896-05

**Authors:** J. McFadden Gaston

**Affiliations:** Atlanta, Ga.


### PERSONAL AND SURGICAL REMINISCENCES OF THE WAR.*

By J. McFADDEN GASTON, M.D.,

ATLANTA, GA.

I was among the first to enter Fort Sumter for the relief of United States soldiers injured in firing a salute before withdrawing from the fort.

I accompanied General Bonham with staff to Richmond and was afterwards at Manassas Junction. Was assigned by General Beauregard as medical director at Manassas. Was called to the advanced post at Fairfax Court House to relieve injuries received in a skirmish with the enemy, and dressed wounds of General Ewell in the shoulder.

I superintended all the preparations for the battle of Manassas, organizing hospitals and appointing medical officers.

Visited battlefields immediately after battle under General Johnston's orders and relieved wounds of both Confederate and of the enemy in like manner.

An interesting incident came under my observation, which has not been reported so far as I know, in finding Lieutenant Blackford with forty prisoners with arms still in hand and a large

*Abstract of the Orator's Address before the Survivors' Association of Ex-Confederate Surgeons, at Spartanburg, S. C., April 22, 1896.

four-horse army wagon in his train. Upon applying to me for advice, he was urged to have his prisoners stack arms in the wagon, and to form in line two and two, which he did.

At my hospital headquarters on the field, a young man, or rather a boy of sixteen, with a large grape-shot in his thigh, very archly asked if he might go back to the field after it was removed.

A case of ligation of the artery of the leg with silver wire occurred in the person of Lieutenant Richardson.

During the afternoon and night following the battle, a large number of surgeons were engaged in operating upon the wounded, and doubtless many amputations were done, which, with farther experience in the war, would not have been considered necessary.

Having half a basketful of legs and arms, I encountered difficulty in getting some one to bury them, and, at last, had to get a negro to dig a hole and bury them.

On the day after the battle, when wounded soldiers were being transported, it was found that Lieutenant De Pass had received a fracture of the skull and required trephining, which was done aboard of the car.

Temporary accommodation of a large number of wounded was secured at Orange Court House, and I proceeded to that point to superintend the further treatment of the wounded.

I recall the fact of seven bullet-holes in a young man, one of which was from a minie-ball imbedded in the clavicle so near the artery that I declined to remove it at that time, and decided to wait until less danger would attend its removal at a later period.

While I was still at Orange Court House Dr. Williams, Dr. Johnston's chief surgeon, returned to Manassas, and sent an order to me to report for duty as surgeon of a Louisiana regiment which was without a surgeon.

Ignoring his authority, I reported to General Beauregard, stating that I was averse to serving as surgeon for the Louisiana regiment, and requesting to be assigned to the South Carolina forces, when I was made brigade surgeon of D. R. Jones, and continued with this brigade under General R. H. Anderson, and was made chief surgeon of his division when General Anderson became major-general.

In the battle of Seven Pines, when going to learn the suitable position from General Anderson for a hospital, I was exposed to the direct fire of the enemy, and beat a hasty retreat.

It was in this action that my two brothers were slain, and I went out at daylight to recover their bodies directly in front of the enemies' lines.

It was in this action that Dr. E. S. Gaillard, on the staff of General Johnston, lost his arm. He afterwards always wrote with his left hand, and was editor and founder of *Gaillard's Medical Journal.*

It often happened that until the opening of a battle, it could not be determined where the battle would take place. This was exemplified in the case of Dr. Post, at the battle of Williamsport, who received a wound of a minie-ball which traversed the top of his head without entering his skull.

After placing him in an ambulance, the rest of the surgeons and myself fell back to a less exposed position. While Dr. A. W. Thompson and myself were riding back, we were overtaken by a shower of bullets, and Dr. Thompson stopped, dismounted, and placed his horse between him and the enemy, but I considered that distance was the best thing to put between me and the firing, and decided to ride on, and the doctor soon came to the same decision.

General Anderson's brother, Captain E. M. Anderson, was killed during this fighting, while near our hospital headquarters, so that it was a timely retreat on our part.

It was here that Dr. W. Moffat Grier, D.D., President of Erskine College at Due West, in this State, and a man now famous in his church, was wounded, and that his leg was amputated by me, doing a circular operation above the ankle.

A very extraordinary lacerated wound was received by a fragment of shell which struck the buttock in the person of Mr. Bratton. A portion of the shell was imbedded in the bottom of this extensive wound, and an opening had been made sufficient to admit one's fist. There was comparatively little shock, and but a small amount of blood lost. The wound was simply approximated with lint and bandage, and left with the direction to use water dressings. I did not see or hear anything more of this patient until about six months ago he presented himself in my office and recounted the interesting circumstances.

Another interesting case that came under my observation at this battle was that of a minie-ball driven into the skull, the base of the ball being imbedded in bone itself, so that it was on a level with the outer surface of the skull. A process was resorted to which has not attracted general attention in surgery, by placing the trephine crown around the base of the ball and cutting out a rim of bone in which it was imbedded, and thus removing the bone with the ball. The cerebral symptoms were removed for the time, but I know nothing of the subsequent history of the case.

In the course of events it was thought proper by the military authorities at Richmond to organize a medical examining board for the examination of medical officers on duty with the different commands. As the different surgeons and assistant surgeons had been commissioned by the authorities, this order met with opposition and great disfavor. The South Carolina brigade, in which I was serving under Brigadier-General D. R. Jones, sent up a protest against its enforcement. Like opposition was manifested by medical officers in other commands. The authorities at Richmond disregarded these manifestations of disapproval on their part, and ordered the examinations to be made.

Having taken an active part in formulating this protest against the examination, I tendered my resignation of my commission as surgeon, advising the other surgeons in the same brigade to accept the conditions and submit to the examination.

My resignation was promptly accepted by the secretary of war, and I was relieved of further duty with the brigade. As I then occupied an independent position, I considered that the government had the right to require any condition for the appointment of surgeons, and made application for a position as surgeon in the Confederate States Army, with the understanding that I would acquiesce in the requirement. This application was acted upon favorably by the secretary of war, and I was directed to present myself for the examination before the board in Richmond. After a formal examination I was approved and reported upon favorably and assigned to duty with D. R. Jones's command, and commissioned under date of original entrance in the service.

The most trying ordeals for the Confederate surgeons were in the seven days battle around Richmond, when they were required to keep up with the movements of the forces and to change their field hospitals from day to day, and underwent more wear and tear than in any other service.

At the battle of Malvern Hill it devolved upon me to perform a high amputation of the thigh in the case of a Federal colonel, the ball shattering the femur. This was satisfactorily performed, but the patient died subsequently of shock.

After withdrawal of the troops from the line of the Chickahominy an order was received from Dr. L. Guild, medical director of the army, to organize a division hospital. I proceeded at once to make requisition for all that was necessary, but was informed that the articles could not be supplied.

Soon after this a patient was recommended by the division board to be sent to Richmond, as they did not have proper facilities for treating him in camp. Upon being submitted to Dr. L. Guild, he indorsed upon the application the remark that this was a proper case for the division hospital. In reply, without due regard to his being higher in authority, I stated that I was surprised that he should suppose that we had a division hospital. He never forgave me this offense, and took occasion subsequently to make me feel that he recollected it.

On one occasion, on the march when returning from Gettysburg, he thought that he had detected that I had been guilty of failure to perform my duty, and took occasion to reprimand me through official orders. I drew General Anderson's attention to this. He asked me if I could make a satisfactory explanation of my conduct. I stated that I could, and did so. Upon this statement General Anderson said that "It seems that the medical director of the army is more disposed to take exception to the chief surgeon of my division than to ascertain the wants of the service, or than he is to adopt measures for their relief."

In this connection I may say that after the battle of Gettysburg Dr. Guild called me to account for leaving my sick and wounded within the enemy's lines. I had selected the college building for our hospital when this building was still in the possession of our forces and in our lines, and sent provisions and nurses and hospital facilities to them when the hospital was out of our lines.

I told Dr. Guild that I had no means of knowing that this position would be abandoned by General Lee, and that my sick and wounded were better provided for than any other portion of the army, as all the hospitals with sick and wounded were now to be left in the hands of the enemy. In a conversation in regard to the administration of the work I had done, Dr. Powell, A. P. Hill's corps surgeon, who was my ranking officer, stated that he had observed on frequent occasions Dr. Guild's criticism upon my performance of duty when he found nothing exceptionable, and he could not understand why this should be until he was informed of this occurrence relating to a division hospital.

Realizing that the antagonism of the medical director might finally get me into trouble, as I might not at all times be free from cause for censure, I notified General Anderson of my desire to avail myself of the first opportunity to be transferred to some other branch of the service. Such an occasion was presented in my being ordered by the surgeon-general when I was absent in South Carolina on sick leave, to go to the relief of the medical department after the battle of Chickamauga. Dr. S. H. Stout, Medical Director of Hospitals, expressed a wish to have me assigned as medical inspector of hospitals in his department, and made an application accordingly.

Upon my return to the division I sent up an application requesting that I be relieved of my duty with General Anderson's division, and be assigned to hospital duty, with the expectation that this understanding would be carried out.

But upon receiving the order for my relief from duty with General Anderson's division, I was directed to report for orders to General Beauregard at Charleston.

But upon doing so, I was instructed by his medical director to proceed to Fort Gaines, Ga., and to organize a general hospital, in view of a probable invasion from Florida by way of the sea. It turned out that the apprehension was unfounded, and hence no patients ever came from that direction.

One of the most pleasing features of my separation with General Anderson and his division was the receipt of the following communication :

HEADQUARTERS ANDERSON'S DIVISION, December 12, 1863.

*Surgeon J. McF. Gaston, formerly Chief Surgeon Anderson's Division :*

SIR :—We, the senior surgeons of brigades of your old division, did not fully realize your transfer from the field until we had read your very kind note of the 9th instant.

We all regret that we did not have the pleasure of shaking you by the hand, and bidding you God-speed ere you left us. It is hardly necessary to tell you that we regret the change beyond the capacity of language to express, for that you had won our high esteem and possessed our confidence must have been even to you apparent. Upon the battle-field, without your counsel and generous co-operation, our arrangements will seem incomplete. You will be missed as the courteous presiding officer of the Board as well as the genial gentleman and friend.

We rejoice that our efforts to discharge our duties have met your commendation ; to continue efficient and faithful we consider, without compliment, that we have but to emulate your high example.

In your new field, Doctor, we wish you all success, and predict a happy and an honored future, such a one as eminent capacity and lofty moral standard alone can win.

We remain with very high respect,

Your friends,

M. L. CROFT,
Senior Surgeon Pose's Brigade.
W. TAYLOR,
Senior Surgeon Wilcox's (old) Brigade.
M. L. THOMAS,
Senior Surgeon Perry's Brigade.
ALF. W. POWELL,
Senior Surgeon Mahone's Brigade.
SAMPSON POPE,
Senior Surgeon Wright's Brigade.

The hospital under my charge at Fort Gaines became the recipient of the hospitals at Macon and other points, to which the wounded were sent from the operations around Atlanta.

I recall a wounded Federal prisoner who was sent back with a secondary trouble of the knee-joint from a bullet wound in that vicinity. The condition of this patient would have warranted an amputation of the leg above the knee, but I refrained from doing so, from the impression that it might be supposed that this extreme measure was resorted to because he was a prisoner.

He died and a somewhat extensive post-mortem examination was subsequently made, which well-nigh involved me in trouble at the close of the war. A Federal officer who was placed on duty at Fort Gaines investigated the facts and sought my whereabouts. But it so turned out that I was then in a foreign land, and no further investigation was made after my return.

After the advance of General Hood into Tennessee, I received orders to break up my hospital and to accompany his movements. But upon reaching Selma, Ala., I received orders to stop at that point, and from there was directed back to Fort Valley. The scope of the work there was very much such as it had been at Fort Gaines.

When the Federal commander, General Wilson, took possession of Macon, I took the responsibility of removing my hospital to Albany, so as to be out of harm's way.

With all the officials and attendants of the hospital, we stopped over at Smithville at the hour of dinner. Our stores were all nailed up and inaccessible, and nobody had any money with which to buy a meal at the hotel. I informed the proprietor of the hotel of our predicament, and offered to barter our spades, shovels, and pickaxes for a meal's victuals; and he acceded to this proposition. We all enjoyed a most substantial repast. I subsequently visited General Wilson at Macon, and reported that I had a hospital outfit with some utensils and stores at Albany.

After inquiry as to the character of the articles, he told me that I could retain them for distribution among the needy people of the country.

A lot of good, substantial clothing was sent to Dawson for deposit, and was left at the depot. Upon passing there subsequently I found that the squad of Federal soldiers stationed there had been taking liberties with these clothes, and I called them together and spoke to them peremptorily that, by the authority of General Wilson, they were commanded to leave these clothes unmolested. They were never troubled, and were distributed among the people.

In a conference with Dr. Frank Hawthorne, who had been in charge of a hospital in South Georgia, it was determined that the South no longer offered favorable conditions for a home, and that we would seek a refuge in Brazil, South America. It was planned that we would meet on a fixed day at New Orleans, each selecting his own route for reaching that point. As pecuniary resources were very limited, and I had to avail myself of a loan of two hundred dollars from a friend, I set about making all the necessary arrangements and, with a well-stocked mess-chest, boarded a steamer on the Chattahoochee, at Fort Gaines.

I was transferred from a river steamer to an ocean steamer at Appalachicola, and reached Mobile bay, where I was landed on the coast, and there awaited a steamer for New Orleans.

At the same point I was fortunate in meeting several Federal negro soldiers, who showed me a great spirit of accommodation in carrying my heavy mess-chest across a neck of land to reach the New Orleans steamer. Without their assistance it would have been utterly impossible to have secured any one to have moved this heavy mess-chest, and knowing, as they did, that I had been a Confederate officer, they rendered me service with alacrity, refusing compensation. With the delay, I reached New Orleans after the date fixed with Dr. Hawthorne, and found that he had gone on to New York.

I succeeded in getting free transportation on a vessel which was to transport troops from New Orleans to New York. It devolved upon me to furnish my own provisions, and my mess-chest was well supplied for this purpose, and was shared by another Confederate, Mr. Charles Mallory, whose resources were worse even than my own.

There were several cases of intermittents among the soldiers, and when it was learned that I was a physician they called upon me for advice. But when it became known that I proposed to give thirty grains of quinine and a half grain of morphine at one dose, immediately before the chill, it caused the officers to doubt my good faith in providing for the welfare of their troops. Upon my assurance that I had repeatedly given this dose with good results always, they submitted to it, and it was attended with complete relief in every case. A fracture of the leg, among the soldiers, occurred from sliding of the door of the hatchway, and crushing the leg. This was dressed with such splints as I could extemporize aboard and was progressing very favorably when we reached New York. I also attended the families of officers who were aboard. I had a unique experience in having my coffee prepared by the cook. It was found that instead of giving me the genuine coffee which was furnished, a substitute was used in preparing my coffee. Upon remonstrating upon this sleight of hand process, they refused to prepare the coffee at all, and ultimately gave me notice that I could only have access to my mess-chest once a day. The steward rendered my situation so unpleasant in every respect that I was extremely exasperated and seriously meditated pitching him overboard. Eventually taking a practical view of the situation, I appealed to the captain, who ordered the steward to allow me every privilege.

My relations with the officers and passengers aboard of the vessel were of the most pleasant nature, although they had fought on a different side, and the following testimonial has been preserved as a souvenir of my professional service:

U. S. TRANSPORT H. S. HAGAR,
NEW YORK HARBOR, July 20, 1865.

We, the undersigned passengers and officers commanding troops on board the U. S. transport H. S. Hagar, hereby respectfully tender our thanks to Dr. J. McF. Gaston, late surgeon of the Confederate army, for his unremitting care for and humane and skillful treatment of the sick of our respective commands during the trip from New Orleans to New York.

Very respectfully,
IRVING D. SOUTHWORTH,
Captain 25th New York Battalion.
ROBERT C. NICHOLS,
First Lieutenant 13th Massachusetts Battalion, Commanding.

*To Dr. J. McF. Gaston, late Surgeon Confederate Army.*

This was a peace-offering of the victor to the vanquished and was accepted as an omen of speedy reconciliation between the soldiers of the North and the South. It is true that political reconstruction was slow in materializing, but since the words of brotherly love, written by Henry Grady, the work of restoration has been progressing most satisfactorily. We have noticed especially the growing confidence amongst the medical men of different sections, and the membership of the different societies is made up from all the various regions of our extensive country.

The American Medical Association and the American Academy of Medicine are to hold their annual meetings at an early day in Atlanta, which opens her doors to all who come, and I would close this task by an invitation to the medical men of South Carolina to come and participate in the scientific work of these societies and partake of the good cheer which will be offered by the profession to all our colleagues who may accept of our hospitality on that occasion.

